# Effect of Training on the Reliability of Satiety Evaluation and Use of Trained Panellists to Determine the Satiety Effect of Dietary Fibre: A Randomised Controlled Trial

**DOI:** 10.1371/journal.pone.0126202

**Published:** 2015-05-15

**Authors:** Vicky A. Solah, Xingqiong Meng, Simon Wood, Roland J. Gahler, Deborah A. Kerr, Anthony P. James, Sebely Pal, Haelee K. Fenton, Stuart K. Johnson

**Affiliations:** 1 Nutrition, Dietetics and Food Science, School of Public Health, Faculty of Health Sciences, Curtin University, Perth, WA, Australia; 2 Food, Nutrition and Health Program, University of British Columbia, Vancouver, BC, Canada; 3 InovoBiologic Inc., Calgary, AB, Canada; 4 Factors Group R & D, Burnaby, BC, Canada; 5 Flinders Centre for Innovation in Cancer, Flinders University, Adelaide, Australia; TNO, NETHERLANDS

## Abstract

**Background:**

The assessment of satiety effects on foods is commonly performed by untrained volunteers marking their perceived hunger or fullness on line scales, marked with pre-set descriptors. The lack of reproducibility of satiety measurement using this approach however results in the tool being unable to distinguish between foods that have small, but possibly important, differences in their satiety effects. An alternate approach is used in sensory evaluation; panellists can be trained in the correct use of the assessment line-scale and brought to consensus on the meanings of descriptors used for food quality attributes to improve the panel reliability. The effect of training on the reliability of a satiety panel has not previously been reported.

**Method:**

In a randomised controlled parallel intervention, the effect of training in the correct use of a satiety labelled magnitude scale (LMS) was assessed versus no-training. The test-retest precision and reliability of two hour postprandial satiety evaluation after consumption of a standard breakfast was compared. The trained panel then compared the satiety effect of two breakfast meals containing either a viscous or a non-viscous dietary fibre in a crossover trial.

**Results:**

A subgroup of the 23 panellists (n = 5) improved their test re-test precision after training. Panel satiety area under the curve, “after the training” intervention was significantly different to “before training” (p < 0.001). Reliability of the panel determined by intraclass correlation (ICC) of test and retest showed improved strength of the correlation from 0.70 pre-intervention to 0.95 post intervention. The trained “satiety expert panel” determined that a standard breakfast with 5g of viscous fibre gave significantly higher satiety than with 5g non-viscous fibre (area under curve (AUC) of 478.2, 334.4 respectively) (p ≤ 0.002).

**Conclusion:**

Training reduced between panellist variability. The improved strength of test-retest ICC as a result of the training intervention suggests that training satiety panellists can improve the discriminating power of satiety evaluation.

## Introduction

Successful strategies to limit the overconsumption of food and beverages are needed to address the rise in incidence of overweight and obesity worldwide. Satiety is the inhibition of eating which occurs as a consequence of having eaten [1, 2]. Specifically, satiety involves an individual recognizing hunger and the feeling of being full after a meal and using this perception to control food intake [3–5]. The effect of foods on satiety has potential importance in the control of overeating since highly satiating food may increase the time in between eating occasions, leading to a reduction in overall energy intake [1,3,6–10].

The most common experimental approach for measuring satiety involves fasted subjects consuming the test food or meal and rating their feeling of fullness or hunger. Fullness and hunger in the fasted state and at prescribed time intervals post-meal are recorded on a line scale marked with descriptive anchors. The panellists’ line scale markings are enumerated by their distance from the start or mid-point [11,12] on the line scale and this score is plotted against postprandial time to generate a postprandial satiety response curve. The area under this curve (AUC) can be used as a summary value to quantify the individual panellist’s satiety response. In addition, the mean value of the AUC of all panellists can then be used as a total satiety response to the experimental food or meal [13]. Researchers routinely use a visual analogue scale (VAS) in satiety studies though awareness of the multifactorial nature of satiety [14]. Raben et al [14] reported measuring hunger sensations under certain circumstances to be a reliable measure with many researchers stating the 100mm VAS satisfactory but suggested variation was due to methodological and biological day to day variability. Raben et al [14] also reported researchers could infer the extent of variability from fasting scores. Similarly, Horner et al [15] found mean postprandial results less variable than fasting results.

Knowledge of the factors that contribute to panellist’ variability would be of value to satiety research. Individuals differ widely in their self-evaluation of the satiety effect of the same meal using line-scales [16] since they are not accustomed to differentiating and scoring the intensity of perceptions such as hunger and fullness that are encountered in daily life. In addition, the expected satiety from a familiar meal may influence the decisions about meal size, as personal perception is determined by experience [17,18]. The satiety effect reported for a meal can change in response to external factors such as anticipation of food, emotions, time of day, level of hunger and proximity to food [19, 20]. Therefore efforts to carefully control factors which influence satiety are needed when designing satiety studies to ensure adequate precision [20]. Reliability, which includes precision, relates the degree of consistency of repeated measurements. High reliability is important in all studies using panellists and has been highlighted as very important in sensory evaluation [21,22].

The ICC provides a statistical measure of reliability that can be used to monitor and assess panels and panellists. The ICC compares ratings by a single panellist to total panel rating variation and quantifies the strength of correlation between panellists [21]. Only when precision and reliability are controlled can the satiety responses of the different foods or meals under evaluation be confidently quantified and differentiated. Reliability of panels for the sensory evaluation of foods has been reported in the literature [21] but that of satiety panels is limited [14, 15, 23].

Reproducibility is defined as test-retest reliability and experts suggest that VAS are ideal for use within subject repeated measure designs [23]. Aspects that may limit the power of the line scales used in satiety assessment to differentiate the satiety effects of foods include the floor/ceiling effect [24, 25] where panellists rate their hunger or fullness as the lowest or highest possible point on the line scale and consequently, greater or lesser hunger/fullness cannot be assessed. Panellists can also cluster their responses at the anchors (descriptors) on line scales [26] which may limit the ability of a line-scale as a tool to differentiate between the satiety effects of different foods. Both floor/ceiling effects and clustering may reduce the ability of satiety studies to detect small, but significant differences between the satiety effects of different foods, although it is important not to limit the usage of the scale if panellist feel the extreme is where there level of fullness or hunger is best described [27, 28].

Training of panellists in the use of line-scales is commonly used in sensory evaluation of foods in order to increase the reliability of the panel to describe attributes of foods [29, 30]. Training approaches used for sensory panels include reaching consensus on the meaning of the descriptors on the line scale (for example when describing a quality parameter such texture or colour) and also orientation training where the panellists become familiar with using the use of line scale [26]. Trained panellists have previously been used to measure the satiety effects of alginate and whey protein-based foods [12]. In a study by Solah et al [12], training involved consumption of a standard meal by fasted panellists who then completed a VAS and subsequently reached a consensus on the meaning of the VAS descriptors. It was concluded however that the effect of training on the precision and reliability of a satiety panel and the benefits of training a satiety panel need further investigation.

The primary outcome measure of satiety research is postprandial satiety response measured by a labelled magnitude satiety line scale (LMS).The primary aim of this study was to investigate if training satiety panellists in the interpretation and use of the satiety line-scale could improve the test-retest precision and reliability of satiety response to a standard breakfast meal using a parallel intervention of either (i) training or (ii) no-training. A secondary aim was to select a “satiety expert panel” from the trained panel to compare the satiety effect of two breakfast meals containing either a viscous or a non-viscous dietary fibre.

## Materials and Methods

### Study 1—The effect of training on the reliability of satiety evaluation

#### Subjects

Twenty four healthy subjects (17 females and 7 males; BMI 18.1–29.5 kg/m^2^), aged 19 to 53 years were recruited from the staff and student population of Curtin University (Perth, Australia) over the period September 2, 2013 to May 30, 2014. The study was approved by the Curtin University Human Research Ethics Committee on August 14, 2013 and informed written consent was obtained from all subjects before the start of the study. The study was retrospectively registered with Australian New Zealand Clinical Trials Registry (ANZCTR) after the enrolment of participants. Curtin University satiety studies that do not involve blood collection are not routinely registered as clinical trials, however a second related study on postprandial glycemia and satiety entailed clinical trial registration. The clinical trial application was submitted for the combined research. In response however, ANZCTR requested this training study and the second satiety study be separate applications. Despite, the delay in ANZCTR registration, the authors confirm that all ongoing and related trials for this drug/intervention are registered.

The exclusion criteria used for this research were individuals that: were smokers; pregnant women; taking medication known to affect satiety; had food allergies; were unwilling to avoid alcohol during the study; normally consumed more than three standard alcoholic drinks per day or; had any history of diabetes, gastrointestinal or cardiovascular diseases. Subjects had not previously participated in a satiety panel.

Participants completed the Three-Factor Eating Questionnaire to evaluate the level of dietary restraint, disinhibition and perceived hunger [31]. The final questionnaire consisting of 51 items was used. Answers were numerically coded [31]. Data analysis was conducted using Statistical Package for Social Sciences, Version 19.0 (IBM Corp Armonk, NY). Selected subjects were required to have low scores of < 10.

A double blind approach was used, with both participant and administrator unaware of test or placebo group allocation. The researcher who determined the subject was eligible for inclusion in the study was unaware to which group the subject would be allocated. Allocation was concealed using a central computer for generation of random codes and each subject was allocated a three digit code, at this point. Subjects maintained their habitual exercise for the entire duration of the study.

#### Experimental design

**Fig 1:**
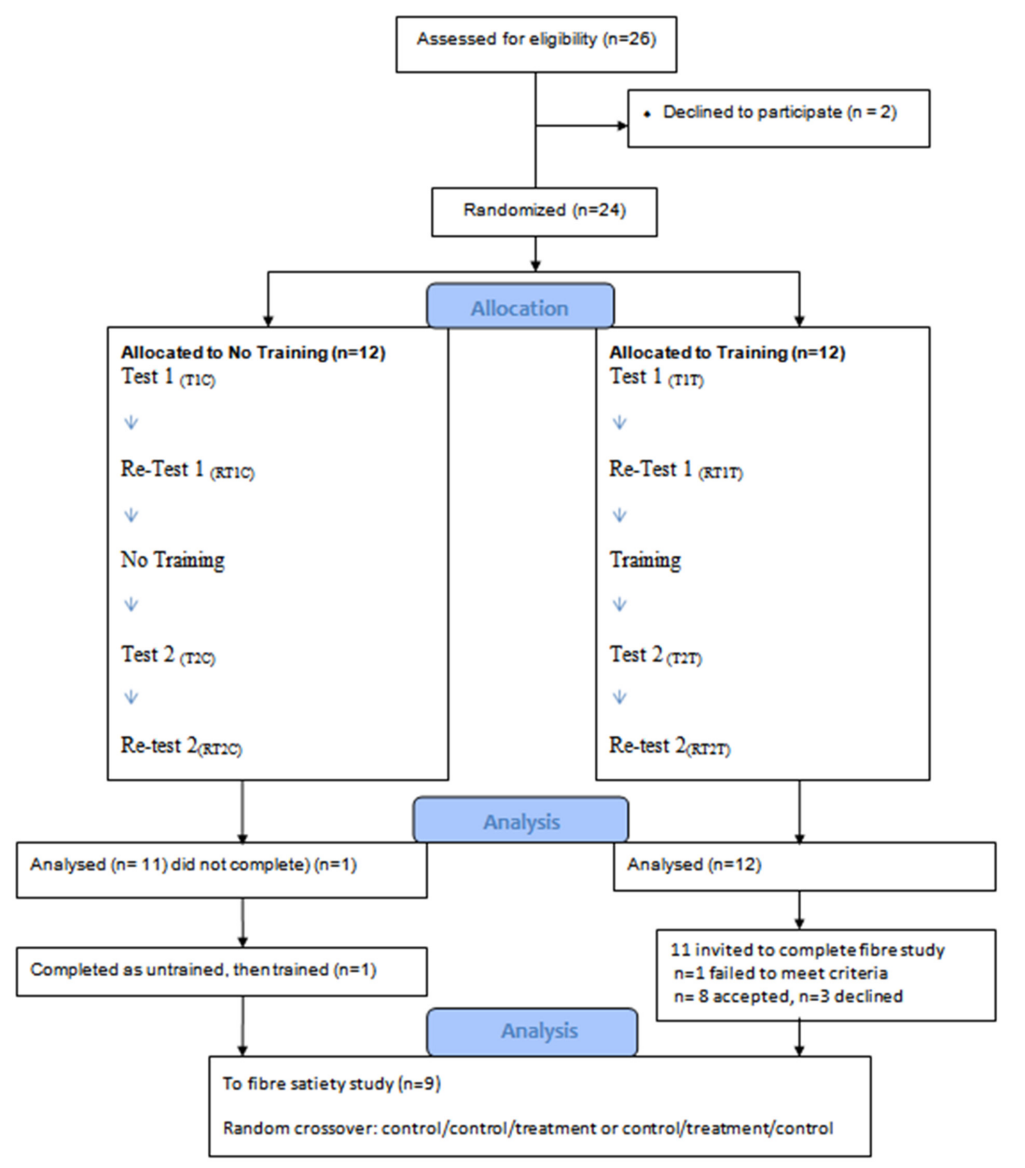
Experimental design: Training and No-training (control), and Fibre satiety study.

This study was a parallel intervention (pre-post-test) (Fig 1) evaluating the satiety of a standard breakfast. Participants were randomly allocated into one of two treatment groups: one to receive training (12 subjects) and the other a control group that would receive no training (12 subjects) (Table 1). Subjects were allocated a computer generated three digit code. There was no significant difference in the characteristics of the control and training group (Table 1).

**Table 1:** Subject (panellists) characteristics.

	No-training (n = 11)	Training (n = 12)
Gender	8 F, 3 M	8 F, 4 M
Age (y)^a^	28.8 ± 14	26.5 ± 9
Weight (kg)^a^	63.5 ± 12.3	66.8 ± 12.5
BMI (kg/m^2^)^a^	23.1 ± 4.3	23.8 ± 5.7

^a^No significant difference between no-training and training (p < 0.05)

Each group consumed the standard breakfast and rated satiety over four consumption occasions, each one week apart. The control group completed the first test-retest (t1C and rt1C, Weeks 1 and 2) of the satiety evaluation of the standard breakfast, then a week later proceeded to the second test-retest (t2C and rt2C, Weeks 3 and 4) without a training session (Fig 1). The training group underwent the training protocol after the first test-retest (t1T and rt1T, Weeks 1 and 2), followed by the second test-retest seven days later (t2T and rt2T, Weeks 3 and 4) (Fig 1).

#### Satiety line scale

A 190mm (19cm) LMS scale was selected for the evaluation of satiety [32] in this study and descriptors provided the basis for definitions used during training (Table 2). The LMS was considered to provide better discrimination of satiety sensations compared to a VAS [32].

**Table 2:** Training LMS descriptors with definitions.

GIH	MH	SH	NHNF	SF	MF	VF	EF	GIF
Too weak to move	Need food	Planning to eat	No desire to eat	Starting to feel full	Feeling full	Do not want to eat more	Cannot eat another thing	Cannot move (food coma)
Grumpy	Stomach grumbles	Snack would fix	Not thinking about food	Could eat more	Do not need to eat more just now	Full as you can get and remain comfortable	Should not eat anymore	Pain/feel sick
Hunger pain					Not physically full	Physical feeling of fullness- no more room for food		Christmas-lunch full
Light headed / dizzy								About to explode/ burst/ vomit
Desperate need to eat								Disgust for over-eating
Constantly thinking of food								
Headache								

**Fig 2:**
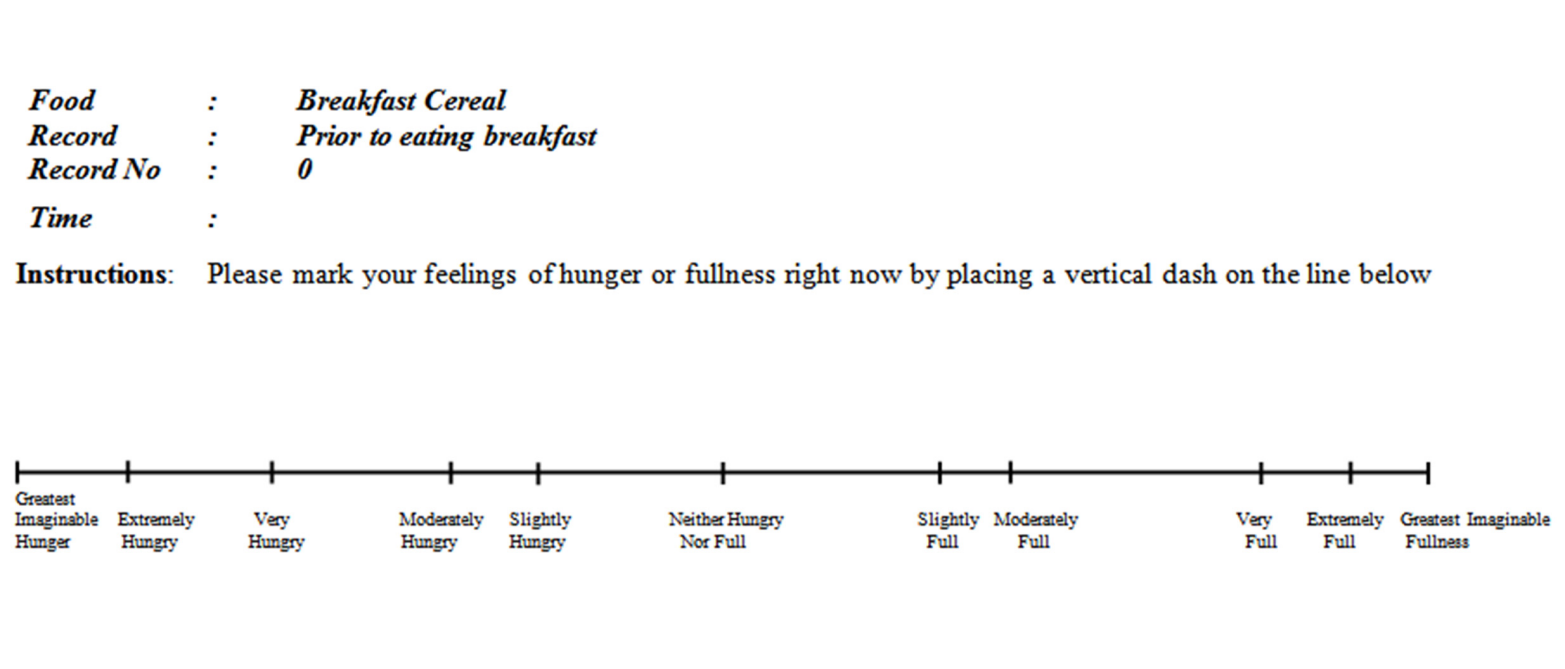
Labelled magnitude satiety scale (LMS).

GIH = Greatest Imaginable Hunger; MH = Moderately Hungry; SH = Slightly Hungry; NHNF = Neither Hungry Nor Full; SL = Slightly Full; MF = Moderately Full; VF = Very Full; EF = Extremely Full; GIF = Greatest Imaginable Fullness.The scale was anchored with words to describe the feeling of hunger from “Greatest Imaginable Hunger” to “Greatest Imaginable Fullness”. The left 95mm (9.5cm) of the LMS had descriptors relating to hunger and right 95mm (9.5cm) of the LMS related to fullness (Fig 2). The line scale marks were enumerated by measuring their distance (mm) from the centre point; therefore a maximum score of 95mm (9.5cm) equated to “Greatest Imaginable Fullness” and minus 95mm (-9.5cm) to “Greatest Imaginable Hunger”.

#### Satiety evaluation protocol

On the evening prior to each testing session, panellists were instructed to fast overnight for 10 hours and consume only water. Prior testing, panellists arrived at the Sensory Evaluation Laboratory at Curtin University and were given instructions about the procedures for the day. Panellists marked their fasting level of hunger/fullness on the LMS scale. Panellists were then requested to consume a standard breakfast meal, in its entirety, within 12 minutes. Panellists were required to mark their feeling of post-meal hunger/fullness on a separate LMS each 15 minutes after the commencement of eating for the first hour and each 30 minutes during the second hour. A postprandial satiety curve was generated for each panellist by plotting their satiety score (mm) against time from start of meal (minutes). The AUC of postprandial satiety was calculated using the trapezoid rule [12].

#### Standard breakfast formulation and composition

The standard breakfast (total weight 228g) contained cereal flakes (15g each of Kellogg’s Branflakes, Cornflakes and Special K), sultanas (8g) and whole milk (175g) with an energy content of 1261kJ [33]. This breakfast was developed by conducting a preliminary satiety evaluation (data not shown) to provide a meal that would induce immediate post-meal satiety of at least “Moderately full” using the LMS in a previously fasted panellist. The nutritional composition of the standard breakfast meal was: protein 11.9g, total fat 6.7g, available carbohydrates 51.8g calculated using the Food Standards Australia and New Zealand Nutrition Panel Calculator [33].

#### Training intervention

The training session of one hour was conducted once during the study with the training group (Fig 1). The training was administered by a primary researcher using a pre-defined scripted protocol. During the training intervention, fasted panellists consumed the standard breakfast meal at time point 0 and completed the LMS. All LMS results were then transcribed on a whiteboard and the results discussed. Consensus was reached by the panellists on the meaning of the descriptor words and more detailed definitions of the descriptors were generated by the panellists and voted upon. The descriptive terms generated served to increase participants’ understanding of the groups accepted meaning of descriptors and thereby decreasing inter panellist variability.

The second aspect of the training was to instruct the panellists on the correct use of the LMS as a continuous scale i.e. to avoid clustering marks at the anchors. During the training session if one panellist voted for “cannot eat another thing” as the definition for “very full” but 11 panellists voted for “cannot eat another thing’ as the definition for “extremely full”, this was accepted by all 12 panellists as the definition to be considered in the satiety study.

#### Data Analysis

All analyses were performed using Stata statistical software (SE 12.1, StataCorp, College Station, TX, USA) and values of p < 0.05 were considered as significant.

Each panellist’s satiety score (n = 23) at each test time point (baseline, 15, 30, 45, 60, 90, 120 minutes), was used to generate postprandial satiety curve for each test occasion and the area under the postprandial satiety curve (AUC) was calculated using the trapezoidal rule. While most satiety studies adjust for baseline, this the data was used as presented by panellists and not adjusted for baseline because fasting satiety scores gave greater within subject variation than postprandial scores, as also reported by Horner et al. [15]. Scores were not adjusted after training in order to maintain the research focus on the post training score.

Intraclass correlation (ICC) was the primary analysis and used to quantify the strength of correlation between panellists [34]. Test-retest reliability was determined using ICC by regression analysis on individual panellist AUC and comparing the variability of AUC at different test and retest occasions of the same panellist to the total variation in AUC across all test and retest occasions and all panellists. ICC was used to reflect the proportion of total variance in difference of AUC that was accounted for by clustering. ICCs were determined from estimation after mixed random effect modeling, in which each subject’s identification code was included as a random effect to account for the intra-group correlation i.e. the lack of independence of measurement within a same subject at different test point, which is the repeated measurement in this case. Using this approach effects of test-retest occasion (pre-intervention = 1 vs post-intervention = 2) and group (training vs no-training) on the ICC of test vs retest AUC was determined.

The mean of individual AUC was calculated and correlation coefficients were calculated to determine if there was a relationship between the panel test occasions (t1vs rt1 and t2 vs rt2).

To determine the effect of training on test-pretest precision, test-retest differences in AUC (t1—rt1; t2—rt2) were first calculated for each panellist. Then a mixed random effect model, was used to identify significant effects of test occasion (pre-intervention vs post-intervention) and group (training vs no-training) on test−retest AUC.

### Study 2—Use of trained panellists to determine the satiety effect of dietary fibre

The aim of training a satiety panel was to assist in planned dietary fibre studies, so it was important to determine if the panel could determine a satiety difference between two dietary fibre products previously studied and as a result inulin and PGX were selected [13].

#### Selection of “Satiety Expert Panel”

Thirteen panellists completed training (twelve from the trained panel and one panellist from the untrained panel who agreed to undergo training). Trained panellists were invited to join the “satiety expert panel” (SEP). Nine panellists accepted, three declined and one panellist from the training intervention failed to meet individual precision criteria.

Individual precision criteria was determined using panellist’s individual score difference t1- rt1 and t2—rt2. Individual scores differences (test-retest) were used in sequential testing for a paired comparison using a modified sensory method for selecting judges by Cross et al [35]. Panellists with score differences between the lower line (L_o_) y = 0.578x - 1.09 and upper line (L_1_) y = 0.578x + 1.09 were considered to have acceptable precision. The first score difference was added to the second score difference etc. The ICC for the “satiety expert panel” was also determined.

#### Evaluation of effect of fibre addition to a breakfast using the “satiety expert panel”

The trained “satiety expert panel” (n = 9) was used to determine if there was a difference in the postprandial satiety effect between a non-viscous control and a viscous fibre, treatment added to a standard breakfast. The primary outcome measure for the SEP study was AUC. Inulin (Fibre Clear, Pharmacy Choice) was selected as the non-viscous fibre control and PolyGlycopleX (PGX) fibre was selected as the viscous fibre treatment. The study design was a blind randomised crossover, with each panellist taking the control breakfast twice and the treatment breakfast once. Panellists were assigned a three digit number and randomly assigned to either control/control/treatment or control/treatment/control treatment order.

Following the satiety evaluation protocol described earlier (Study 1), fasted panellists consumed the standard breakfast meal with either 5g of inulin (control) (t1SEP and t2SEP) or 5 g PGX fibre (treatment) (t3SEP) sprinkled on the standard breakfast. Data collection and analysis was performed as described above for Study 1.

#### Palatability

A 150 mm Likert scale was used to assess palatability or ‘liking’. The question “How much did you like this food?” was rated from dislike very much to like very much, where dislike very much = -3, dislike moderately = -2, dislike slightly = -1, neither like nor dislike = 0, like slightly = 1, like moderately = 2 and like very much = 3.

## Results and Discussion

### Study 1—The effect of training on the reliability of satiety evaluation

Twenty three panellists completed Study 1 which comprised of twelve in the training group and eleven in the no-training group.

#### Consensus on descriptor definitions during training

During training, panellists (n = 12) agreed on the definitions for the LMS descriptors (Table 2). The consensus on the meaning of the descriptor words during the training resulted in agreed definitions that were either related to physical and psychological feelings, for example thinking or planning out about food or hunger and fullness (Table 2).

This research aimed to align personal experience of hunger and fullness without losing individual sensitivity. If individual postprandial satiety responses (within subject after the same meal) are highly variable because panellists are not able to adequately describe their feelings or experiences, training serves to reduce this variation. Trained panellists can describe their feelings of hunger and fullness using a scale in a more reliable way. There is a danger that training may change panellist thinking to be analytical and unnatural. Satiety evaluation however requires that individuals remain sensitive to their own feelings of hunger and fullness, so awareness of being too analytical must be highlighted to panellists during training.

In addition to reaching consensus on the definitions of LMS descriptors, training provided panellists with practice in the use of the LMS [26]. While a drawback of using the LMS is that scale category terms and positions must be selected [36] the LMS provided an ideal tool for training. Appetite research frequently uses an unstructured 100 unit VAS [36] so future research will look at how training using a LMS can be applied to studies that use a VAS.

#### Relationship between the panel test occasions

Measurement of the relationship of trained panellist AUC between test occasions (t1 vs rt1, t2 vs rt2) showed the correlation coefficient was greater *r* = 0.95 after training compared to *r* = 0.7 prior to training (p < 0.001).

The no-training panellist’s AUC correlation coefficient was also greater from *r* = 0.66 (t1 vs rt2) to *r* = 0.8 (t2 vs rt2)(p < 0.01) most likely due to having the experience of using the LMS therefore a better understanding. Hayes et al [26] states that panellist experience is known to improve the use of line scales. This finding shows the importance of orientation training for a satiety panel in the use of the LMS or line scale.

The correlation between test occasions for the trained group was significantly higher than the no-training group (p < 0.001) highlighting the importance of training.

#### Effect of training on overall test retest precision of panel

**Fig 3:**
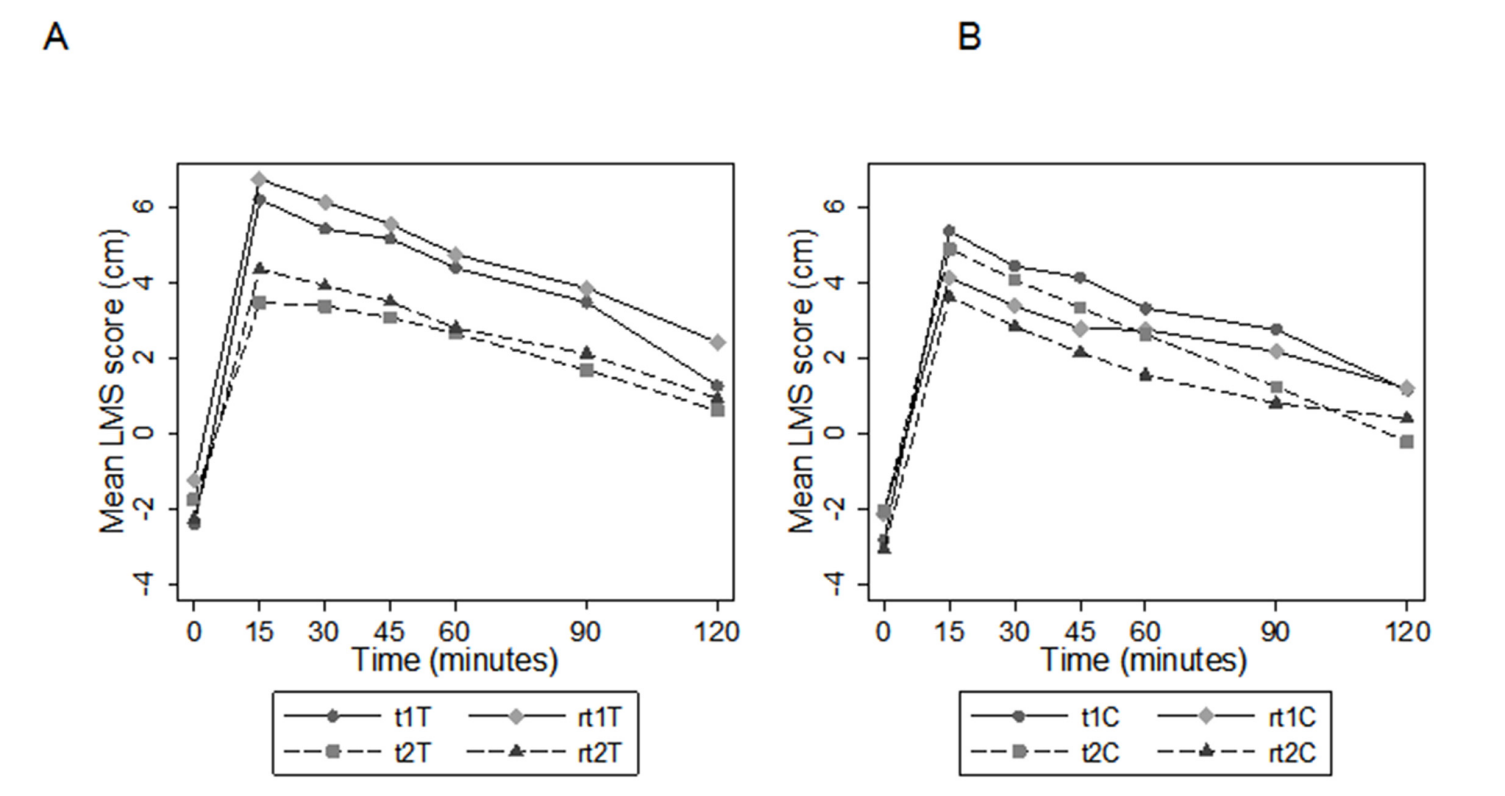
Post-prandial satiety responses (satiety score in cm over time) for each test and retest occasion (See Fig 1): A, training group (n = 12); B, no-training group (n = 11).

Fig 3 presents the mean of postprandial satiety scores for each test and retest occasion for the training group (Fig 3A) and the no-training group (Fig 3B). The AUC of the satiety responses is presented in Table 3. The use of these AUC in the calculation of test-retest precision is given in Table 3. The precision of the AUC was not affected by the intervention (Table 3, (rt2—t2) − (rt1—t)) in either the training group (*p* = 0.66) or the no-training group (*p* = 0.83). In addition, there was no difference in the effect of intervention (Table 3, (rt2—t2) − (rt1—t) between the two groups (treatment vs control) (p = 0.70). It is worthy of note however that the second test-retest mean (rt2—t2) was different to the first (rt1—t).The results show that test-retest precision of trained panel would reduce panel variability in a satiety study though the AUC may be relatively high due to the influence of several panellists’ ceiling effect.

**Table 3:** The area under the curve (AUC) of the satiety response at each test-retest occasion segregated by treatment (training or no-training).

**A**	**C (No-training)**	**T (Training)**	**Between group difference**
	**AUC (cm.min)**		**(T vs C)**
	Mean (SD)	Mean (SD)	Mean difference (SE)
t1	364.5 (319.8)	457.4 (269.3)	92.9 (122.1)
rt1	263.4 (274.7)	527.7 (244.2)	264.2 (107.0)
t2	243.7 (317.1)	257.3 (295.7)	13.6 (126.0)
rt2	164.4 (317.9)	301.6 (292.3)	137.1 (125.6)
**B**	**C (No-training)**	**T (Training)**	**Between group difference**
	**AUC (cm.min)**	**(T vs C)**	
Change	Mean (SE), p value	Mean (SE), p value	Mean difference (SE), p value
(t1− rt1)	-101.1 (89.6), p = 0.26	70.2 (57.6), *p* = 0.22	171.3 (110.9), p = 0.12
(t2− rt2)	-79.3 (59.7), p = 0.19	44.3 (26.5), p = 0.10	123.5 (69.4), p = 0.08
Difference (rt2 − t2) − (rt1 − t1)	21.8 (100.9), p = 0.83	-26.0 (59.1), p = 0.66	-47.8 (122.4), p = 0.70

A. Mean difference between treatments at each test or re-test occasion. B. Differences in test minus retest AUC by treatment (training vs no-training) and by test-retest occasion (pre-training = 1, post-training = 2).

#### Effect of training on ceiling effect

The AUC for the training group (Table 3) was significantly lower post-intervention than pre-intervention: t2−t1 = -200.1, p = 0.005; rt2−rt1 = -226.1, p = 0.003. In the no-training group however there were no significant differences between the pre- and post-intervention AUC (p > 0.05).

The lower AUC of the standard meal after training indicates that the “ceiling effect” was reduced. This suggest that the training protocol used in the present study should make the LMS scale more applicable for differentiating the satiety effects of very highly satiating foods.

The difference between test two (t2) (using average values of t2 and rt2) and test one t1 (average values of t1 and rt1) was -213.1 (SE = 48.2) for the trained group (p < 0.001), and -109.9 (SE = 54.4) for the non-trained group (p = 0.043).

#### Effect of training on reliability of panel

Pre-intervention, the test-retest ICC for satiety AUC of the training group was 0.70 (95% CI; 0.35, 0.91) compared with the no-training group’s AUC of 0.36 (95% CI; 0.07, 0.81). Post-intervention, the ICC for the trained group was 0.95 (95% CI; 0.85, 0.98) while that for the no-training group was 0.75 (95% CI; 0.46, 0.91).

There was an increase in the strength of correlation, ICC for the trained group compared to the no-training group. These results indicate that training increased the reliability of the satiety panel and hence training of panellists is likely to increase their power to discriminate between differences in satiety effects of foods. These results suggest that smaller numbers of trained panellists as opposed to larger numbers of un-trained panellists would be needed to obtain the same discriminatory power.

#### Individual test precision

Results from Study 1 showed that prior to intervention, individual panellists had a mean score difference (rt1C − t1C or rt1T − t1T) in the range of -3.2 to 4.7. Prior to intervention, 11 of the 23 panellists retest difference was not significantly different (p < 0.05) to the mean value of the other panellists. Five of the 12 the panellists in the Study 1 training group had test-retest differences that were significantly different (p < 0.05) to that of the other panellists and hence were considered to have non-acceptable test-retest precision. For these five panellists, the after training mean score difference rt2—t2 ranged from -0.01 to -0.5. Post-intervention, 10 of the 12 panellists in the Study 1 training group had lower test-retest score differences (rt2T —t2T) indicating improved test-retest precision. The loss of behavioural flexibility by panellists described by Blundell et al [36] due to training is a possible problem that needs further research.

Untrained panellists mean score difference ranged from -4.0 to 1.8 (rt2C - t2C) although six of the untrained panellists gave precise results without training (-0.07 to 0.07).

Untrained volunteers or inexperienced panellists may be reliable satiety panellists however this research showed that some volunteers had poor test-retest precision and may not be valuable in a satiety panel.

This finding indicates pre-screening of test retest precision could assist in satiety studies.

In conclusion some panellists were precise without training. Training improved panellist precision in those who were not precise prior to training.

#### Palatability

The panellists mean rating of “How much did you like this food?” on the Likert scale was 1.3 ± 1.6 so the panel “liked slightly” to “liked moderately “showing the breakfast meal was palatable.

### Study 2—The use of trained panellists to determine the satiety effect of dietary fibre, inulin and PGX

#### Individual selection of the satiety expert panel

(Eight from the trained panel and one panellist from the untrained panel agreed to undergo training)

Sequential testing of mean scores difference showed the SEP were within the set criteria i.e. between the L_o_ and L_1_ lines.

**Fig 4:**
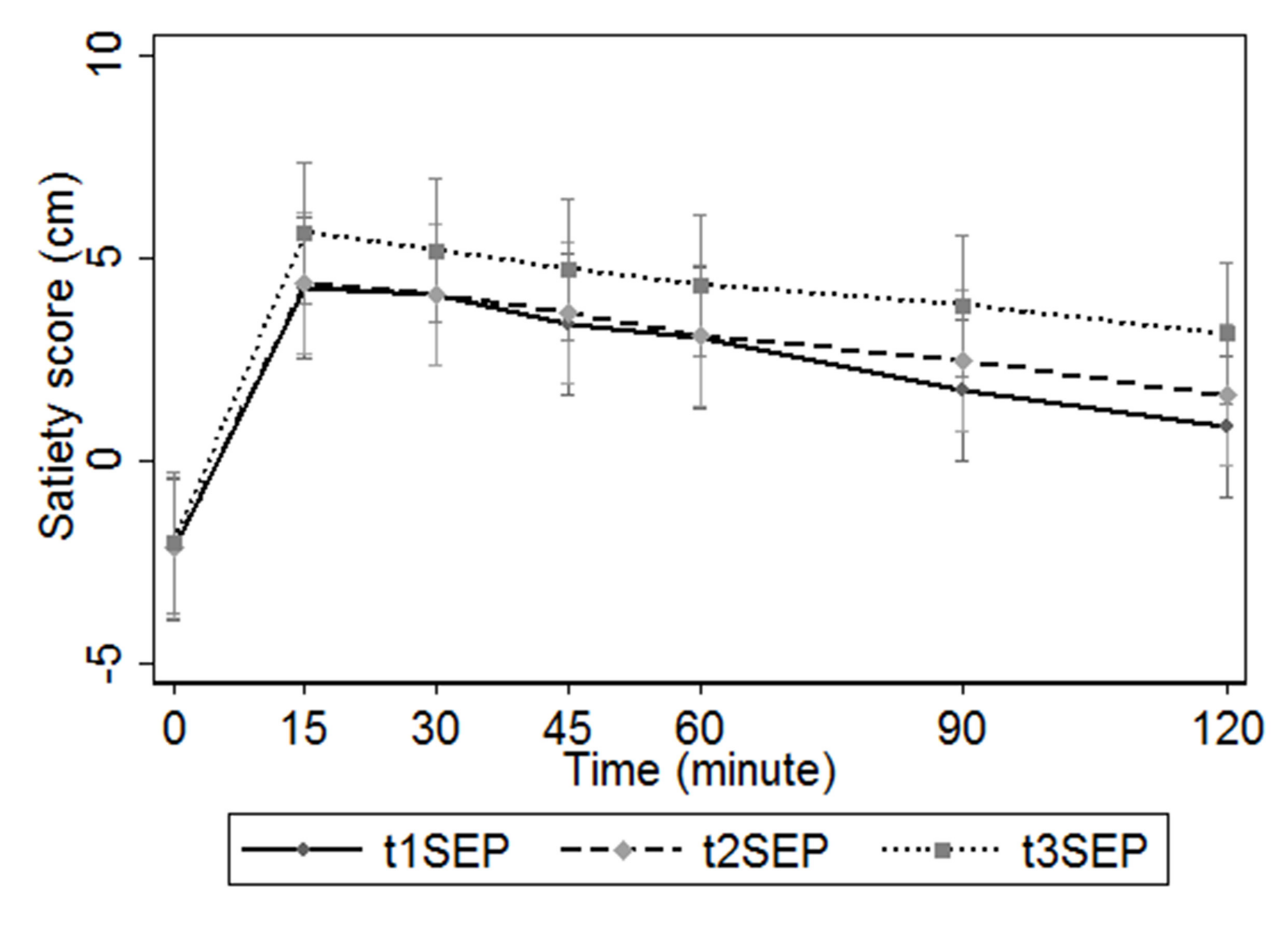
Post-prandial satiety response (satiety score in cm over time) (mean and 95% confidence interval) of the “satiety expert panel” (n = 9). t1 SEP and t2SEP = control (inulin) breakfast; t3SEP = PGX breakfast.

The postprandial satiety response by the SEP to the PGX breakfast (t3SEP) and the control inulin breakfast (t1SEP and t2SEP) is given in Fig 4. Table 4 presents the AUC for these postprandial satiety responses. There was no significant difference in the AUC for the two control breakfast occasions (p = 0.25). The mean satiety response (AUC) for the PGX fibre (treatment) was 478.2 and for the inulin (controls) 291.8 and 334.4.The PGX breakfast gave a significantly higher satiety AUC than the control breakfast (p = 0.002 and p < 0.001) with the mean difference in the AUC between the treatments being 30 to 40%. A 10% difference in AUC is considered a realistic difference for significance [20, 36]. In addition, the trained panel confirmed the PGX increased satiety compared to inulin as reported in previous research [20] and provides a basis for future satiety fibre studies.

**Table 4:** Area under curve (AUC) of postprandial satiety response of “satiety expert panel” (n = 9).

Treatment	Mean AUC (SD/SE)
	(cm.min)
t1SEP	291.8 (331.2)
t2SEP	334.4 (324.6)
t3SEP	478.2 (253.8)
Difference	Mean (SE), *p* value
(t2SEP—t1SEP)	*42*.*7 (22*.*6)*, p = *0*.*10*
(t3SEP—t1SEP)	*186*.*4 (39*.*6)*, p = *0*.*002*
(t3SEP—t2SEP)	*143*.*8 (48*.*5)*, p = *0*.*02*

t1SEP and t2SEP = control (inulin) breakfast; t3SEP = PGX breakfast.

The trained SEP (n = 9) determined that there was a significant difference between two fibres PGX and inulin in their satiety effect (AUC). The use of trained panellists to determine the satiety effect of various dietary fibres will provide further evidence of the effect of fibre on satiety and the benefit of training panellists to satiety research.

## Conclusion

This study evaluated the effect of training on the reliability of satiety evaluation. The panel, after training, provided more reproducible assessment of the postprandial satiety response to a standard breakfast. Training was also found to reduce between panellist variability. This research indicates that the trained panel has the potential to increased discriminatory power to detect differences in the satiety effect of treatments. The ICC, which quantifies the strength of correlation was used as a measure of reliability, and found to increase with training. Panellists were screened for individual precision and a “satiety expert panel (SEP)” selected from those meeting preset precision criteria. This SEP detected a significant difference in the postprandial satiety effect of control breakfast containing non-viscous fibre and a breakfast containing a viscous fibre. Further research is needed to verify if training is as effective with alternate study designs with a different group of trained panellists such as older or obese panellists who may respond differently.

Based on the findings of this study, the assessment of individual’s test re-test precision is highly recommended prior to their inclusion in a satiety panel. Furthermore screening for test-retest precision and conducting the training protocol described in this study is recommended to ensure the most reliable panel. In circumstances when this training program cannot be undertaken, it is recommended that panellists practice in the use of the satiety line scale prior to commencement of a satiety study; as we have demonstrated that this experience can improve test re-test precision. In conclusion, training improved the reliability of the satiety line-scale and using a trained panel should therefore be beneficial in satiety evaluation studies.

## References

[1] Gerstein DE, Woodward-Lopez G, Evans AE, Kelsey K, Drewnowski A. Clarifying concepts about macronutrients' effects on satiation and satiety. J Am Diet Assoc. 2004; 104: 1151–1153. 15215775 10.1016/j.jada.2004.04.027

[2] Slavin JL. Dietary fiber and body weight. Nutrition. 2005; 21: 411–418. 15797686 10.1016/j.nut.2004.08.018

[3] Benelam B. Satiation, satiety and their effects on eating behaviour. Nutr Bull. 2009; 34: 126–173.

[4] Flood-Obbagy JE, Rolls BJ. The effect of fruit in different forms on energy intake and satiety at a meal. Appetite. 2009; 52: 416–422. 10.1016/j.appet.2008.12.001 19110020 PMC2664987

[5] Mattes RD. Effects of a combination fiber system on appetite and energy intake in overweight humans. Physiol Behav. 2007; 90:705–711. 17292929 10.1016/j.physbeh.2006.12.009

[6] Anderson GH, Moore SE. Dietary proteins in the regulation of food intake and body weight in humans. J Nutr. 2004; 134: 974–979. 15051857 10.1093/jn/134.4.974S

[7] Pal S, Ellis V. The acute effects of four protein meals on insulin, glucose, appetite and energy intake in lean men. Br J Nutr. 2010; 104:1241–1248. 10.1017/S0007114510001911 20456814

[8] Pereira MA, Ludwig DS. Dietary fiber and bodyweight regulation: observations and mechanisms. Pediatr Clin North Am. 2001; 48: 969–980. 11494646 10.1016/s0031-3955(05)70351-5

[9] Reichert RG, Reimer RA, Kacinik V, Pal S, Gahler RJ, Wood S. Meal replacements and fibre supplement as a strategy for weight loss. Proprietary PGX meal replacement and PGX fibre supplement in addition to a calorie-restricted diet to achieve weight loss in a clinical setting. Biotech Genetic Eng Rev. 2013; 29: 221–229. 10.1080/02648725.2013.801229 24568282

[10] Slavin J, Green H. Dietary fibre and satiety. Nutrition Bulletin.2007; 32: 32–42.

[11] Holt SHA, Brand-Miller JC, Stitt PA. The effects of equal-energy portions of different breads on blood glucose levels, feelings of fullness and subsequent food intake. J Am Diet Assoc. 2001; 101:767–773. 11478473 10.1016/S0002-8223(01)00192-4

[12] Solah VA, Kerr D, Meng X, Adikara A, Binns C, Zhu K, et al Differences in satiety effects of alginate and whey protein-based foods. Appetite. 2010; 54: 485–91. 10.1016/j.appet.2010.01.019 20144671

[13] Solah VA, Brand-Miller JC, Atkinson FS, Gahler RJ, Kacinik V, Lyon MR, et al Dose-response effect of a novel functional fibre, PolyGlycopleX, PGX, on satiety. Appetite. 2014; 77: 74–78. 10.1016/j.appet.2014.02.021 24631638

[14] Raben A, Tagliabue A, Astrup A. The reproducibility of subjective appetite scores. Br J Nutrition. 1995; 73: 517–530. 7794869 10.1079/bjn19950056

[15] Horner KM, Byrne NM, King NA. Reproducibility of subjective appetite ratings and ad libitum test meal energy intake in overweight and obese males. Appetite. 2014; 81: 116–122. 10.1016/j.appet.2014.06.025 24953196

[16] Schuring E, Quadt F, Kovacs EM, Meullenet JF, Wiseman S, Mela DJ. A quantitative method for estimating and comparing the duration of human satiety responses: Statistical modeling and application to liquid meal replacers. Appetite. 2012; 59: 601–609. 10.1016/j.appet.2012.07.003 22796948

[17] Brunstrom JM, Shakeshaft NG, Scott-Samuel NE. Measuring ‘expected satiety’ in a range of common foods using a method of constant stimuli. Appetite. 2008; 51: 604–614. 10.1016/j.appet.2008.04.017 18547677

[18] Schifferstein HNJ. Labeled magnitude scale: A critical review. Food Quality Pref. 2012; 26: 151–158.

[19] Bornet FRJ, Jardy-Gennetier A-E, Jacque N, Stowell J. Glycaemic response to foods: Impact on satiety and long-term weight regulation. Appetite. 2007; 49: 535–553. 17610996 10.1016/j.appet.2007.04.006

[20] Flint A, Raben A, Blundell JE, Astrup A. Reproducibility, power and validity of visual analogue scales in assessment of appetite sensations in single test meal studies. In J Obe. 2000; 24: 38–48. 10.1038/sj.ijo.0801083 10702749

[21] Bi J, Kuesten C. Intraclass correlation coefficient (ICC): a framework for monitoring and assessing performance of trained sensory panels and panellists. J Sensory Stud. 2012; 27: 352–364.

[22] Lawless H, Heymann H. Sensory Evaluation of Food: Principles and Practice. New York: Chapman and Hall 1998.

[23] Stubbs R, Hughes D, Johnstone A, Rowley E, Reid C, Elia M, et al The use of visual analogue scales to assess motivation to eat in human subjects: a review of their reliability and validity with an evaluation of new hand-held computerised systems for temporal tracking of appetite ratings. Br J Nut. 2000; 84: 405–415. 10.1017/s0007114500001719 11103211

[24] Bartoshuk LM. Comparing sensory experiences across individuals: recent psychophysical advances illuminate genetic variation in taste perception. Chem Senses. 2000; 25: 447–460. 10944509 10.1093/chemse/25.4.447

[25] Duffy VB, Bartoshuk LM. Food acceptance and genetic variation in taste. J Am Diet Assoc. 2000; 100:647–655. 10863567 10.1016/S0002-8223(00)00191-7

[26] Hayes JE, Allen AL, Bennett SM. Direct comparison of the generalized visual analog scale (gVAS) and general labeled magnitude scale (gLMS). Food Qual Pref. 2013; 28: 36–44. 23175601 10.1016/j.foodqual.2012.07.012 PMC3501107

[27] Cardello AV, Schutz HG. Research note. Numerical scale-point locations for constructing the LAM (labeled affective magnitude) scale. J Sensory Stud. 2004; 19: 341–346.

[28] Cardello A, Lawless HT, Schutz HG. Effects of extreme anchors and interior label spacing on labeled affective magnitude scales. Food Qual Pref. 2008; 19: 473–480.

[29] Lawless HT, Popper R, Kroll BJ. A comparison of the labeled magnitude (LAM) scale, an 11-point category scale and the traditional 9-point hedonic scale. Food Qual Pref. 2010; 21: 4–12.

[30] Kreutzmann S, Thybo AK, Bredie LP. Training of a sensory panel and profiling winter hardy and coloured carrot genotypes. Food Qual Pref. 2007; 18: 482–489.

[31] Stunkard AJ, Messick S. The three-factor eating questionnaire to measure dietary restraint, disinhibition and hunger. J Psychosom Res. 1985; 29: 71–83. 3981480 10.1016/0022-3999(85)90010-8

[32] Zalifah MK, Greenway DR, Caffin NA, D'Arcy BR, Gidley MJ. Application of labelled magnitude satiety scale in a linguistically-diverse population. Food Qual Pref. 2008; 19: 574–578.

[33] Food Standards Australia and New Zealand, Nutrition Panel Calculator. Available: http://www.foodstandards.gov.au.

[34] Ip EH, Wasserman R, Barkin S. Comparison of intraclass correlation coefficient estimates and standard errors between using cross-sectional and repeated measurement data: The Safety Check cluster randomized trial. Contemp Clin Trials; 2011 32: 225–232. 10.1016/j.cct.2010.11.001 21070889 PMC3034823

[35] Cross HR, Moen R, Stanfield MS. Training and testing of judges for sensory analysis of meat quality. Food Tech. 1978; 32: 48–54.

[36] Blundell J, de Graaf C, Hulshof T, Jebb S, Livingstone B, Lluch A, et al Appetite control: methodological aspects of the evaluation of foods. Obes Rev. 2010; 11: 251–270. 10.1111/j.1467-789X.2010.00714.x 20122136 PMC3609405

